# An RpoN-dependent PEP-CTERM gene is involved in floc formation of an *Aquincola tertiaricarbonis* strain

**DOI:** 10.1186/s12866-022-02745-1

**Published:** 2023-01-19

**Authors:** Ming Xia, Dianzhen Yu, Han Chen, Jingcheng Dai, Na Gao, Shuyang Li, Xuezhi Bi, Dongru Qiu

**Affiliations:** 1 School of Life Sciences, Hubei Engineering Research Center for Protection and Utilization of Special Biological Resources in the Hanjiang River Basin, Jianghan University, Wuhan, 430056 China; 2 Hubei Key Laboratory of Environmental and Health Effects of Persistent Toxic Substances, Jianghan University, Wuhan, 430056 China; 3 University of Chinese Academy of Sciences, Beijing, 100049 China; 4 Institute for Nutritional Sciences, SIBS, Chinese Academy of Sciences, Shanghai, 200031 China; 5 Institute of Hydrobiology, Chinese Academy of Sciences, Wuhan, 430072 Hubei Province China; 6 Fisheries Research Institute, Anhui Academy of Agricultural Sciences, Hefei, 230031 China; 7 Bioprocessing Technology Institute, Agency for Science, Technology and Research, Singapore, 138668 Singapore

**Keywords:** Bacterial floc formation, PEP-CTERM proteins, Flocculation, RpoN sigma factor, *Aquincola tertiaricarbonis*

## Abstract

**Background:**

The floc is a characteristic of microbial aggregate growth, displaying cloudy suspensions in water. Floc formation has been demonstrated in a series of bacteria and the floc-forming bacteria play a crucial role in activated sludge (AS) process widely used for municipal sewage and industrial wastewater treatment over a century. It has been demonstrated that some exopolysaccharide *biosynthesis genes and the sigma factor (sigma54 or rpoN) were required for floc forming in some bacteria. However,* the mechanism underlying the floc formation stills need to be elucidated.

**Results:**

*In this study,* we demonstrate that a TPR (Tetratricopeptide repeats) protein-encoding gene *prsT* is required for floc formation of *Aquincola tertiaricarbonis* RN12 and an upstream PEP-CTERM gene (designated *pepA*), regulated by RpoN1, is involved in its floc formation but not swarming motility and biofilm formation. Overexpression of PepA could rescue the floc-forming phenotype of the *rpoN1* mutant by decreasing the released soluble exopolysaccharides and increasing the bound polymers.

**Conclusion:**

*O*ur results indicate that the wide-spread PEP-CTERM proteins play an important role in the self-flocculation of bacterial cells and may be a component of extracellular polymeric substances required for floc-formation.

**Supplementary Information:**

The online version contains supplementary material available at 10.1186/s12866-022-02745-1.

## Background

It is long known that the activated sludge bacteria including *Zoogloea*, *Thauera* and *Pseudoduganella* species are capable of forming flocs in which bacterial cells are self-flocculated by an extracellular matrix composed of secreted extracellular polymeric substances *(EPS)* including exopolysaccharides and other biopolymers [1, 2]. Another floc-forming bacterium, *Aquincola tertiaricarbonis* RN12, was isolated from a Chinese water supply well in which nuisance brown precipitates frequently occurred and cause public concerns among the local inhabitants [3]. Certain *Aquincola* strains are capable of degrading gasoline-related contaminants [4–7]. In addition, *Aquincola* is one of the predominant proteobacteria in a membrane bioreactor (MBR) treating antibiotics-containing wastewater [8]. Since the RN12 strain could readily form flocculent precipitates under laboratory cultivation conditions and could be manipulated genetically, we have used this strain as a model to study the mechanisms underlying the formation of brown precipitates found in a water supply well.

It is believed that bacterial flocs may confer bacterial resistance to the predation of protozoa and other invertebrates [9] and may also be related to other important traits such as denitrification and nitrogen fixation. We have conducted genome sequence and annotation, as well as molecular genetics analysis on the floc-formation of *A. tertiaricarbonis* RN12 strain [3]. A large gene cluster for EPS biosynthesis and a gene encoding the alternative sigma factor RpoN1, one of the four paralogues, are required for the floc-formation of *A. tertiaricarbonis* RN12 strain [3]. The bacterial cells of the isolated mutants are planktonic rather than flocculated. Interestingly, the biosynthesis of exopolysaccharides remained in the rpoN1-disrupted mutant, but most of the EPS was released and dissolved in the culture broth rather than bound to the bulk of cells [3]. Consistently, the transcription of exopolysaccharide biosynthesis genes seems not to be regulated by RpoN1 [3]. These results indicate that RpoN1 may regulate the expression of certain gene(s) involved in the self-flocculation of bacterial cells but not in the biosynthesis and secretion of EPS required for floc-formation.

By using phylogenetic profiling methods, a family of special proteins, termed PEP-CTERM domain containing proteins were found and described as a series of proteins that have a short C-terminal homology domain, including a conserved Pro-Glu-Pro (PEP) motif, and a predicted N-terminal signal peptide for secretion and sorting [10]. All the bacteria encoding these putative PEP-CTERM proteins belong to the gram-negative bacteria with an outer membrane and encoding a gene cluster for production of exopolysaccharide. However, the possible relationships between these proteins and EPS remained largely unknown [10]. Interestingly, a TPR protein-encoding gene (prsT) is frequently present around the PEP-CTERM domain protein gene, whose functions remained to be further studied [10].

In the present study, we have isolated and analyzed more transposon mutants defective in floc formation. It has been shown that a PEP-CTERM gene is regulated by the global transcription regulator RpoN1 and could mediate the floc formation. These results provide insights into the regulation of floc formation and the underlying mechanisms in Aquincola, Zoogloea and other floc-forming bacteria.

## Results and discussion

### Complete genome of *Aquincola tertiaricarbonis* RN12 strain

**Fig. 1:**
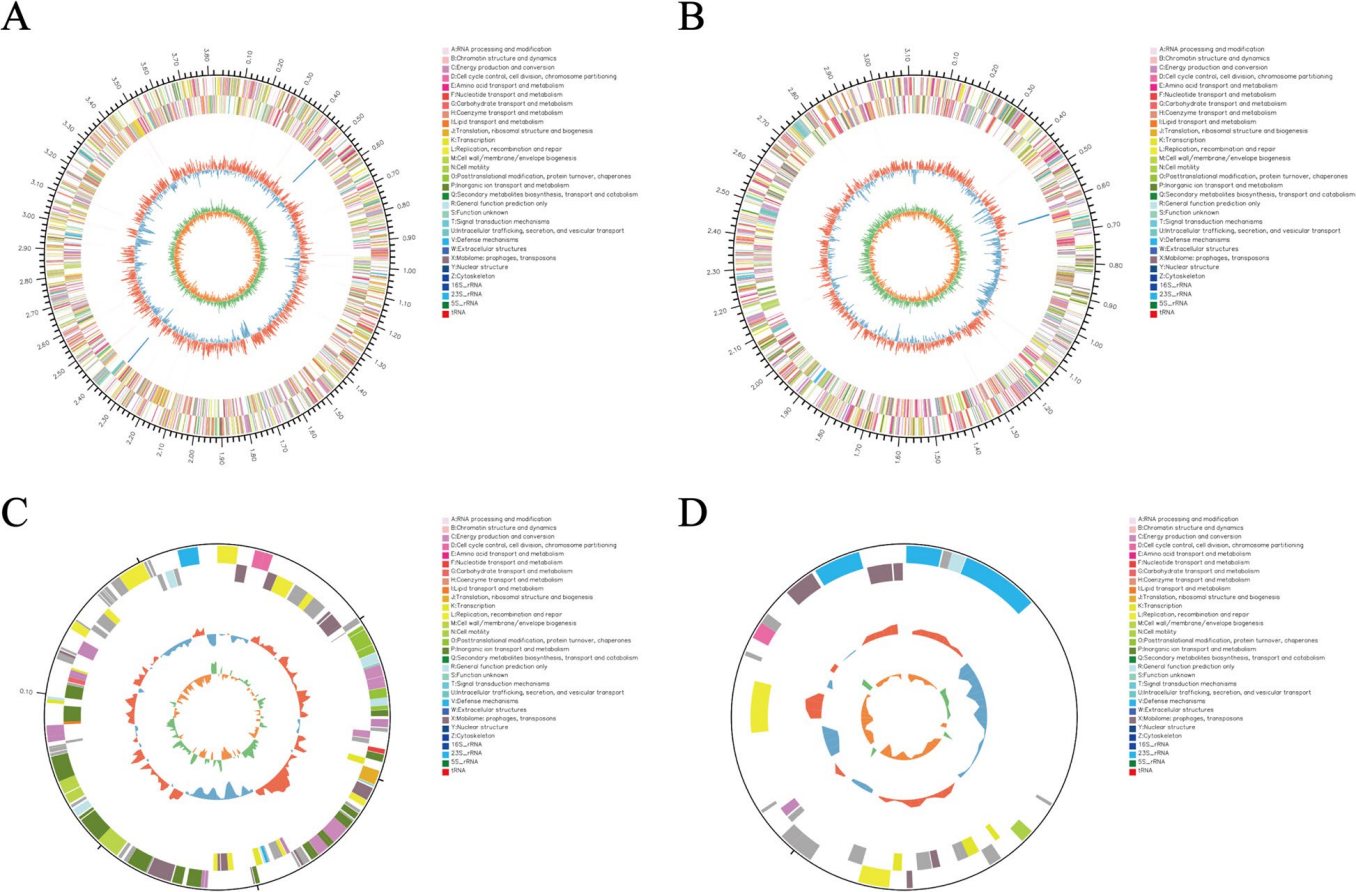
Circular genome maps of the chromosome 1 (**A**), the chromosome 2 (**B**), the plasmid A (**C**), and the plasmid B (**D**) in *Aquincola tertiaricarbonis* RN12 strain, from outside to inside indicate the following: Circle 1: indicator of genome size; Circle 2 and 3: coding sequences in plus and minus strand, different colors represent the functional classification of COG with different CDS; Circle 4: the distribution of rRNA and tRNA; Circle 5: G + C content (the outer circle shown in red is greater than the average content, and the inner circle shown in green is less than the average content); Circle 6: GC skew (green represents the GC skew > 0, orange represents the GC skew < 0)

Both Illumina Hiseq and Pacific Biosciences platforms were used to complete the whole genome sequencing of *Aquincola tertiaricarbonis* RN12 strain. Four double stranded circular DNAs, including two chromosomes, termed chromosome 1 and chromosome 2, and two plasmids, the plasmid A (pA) and the plasmid B (pB), were identified (Table 1 and Fig. 1). The size of chromosome 1 was 3,831,859 base pairs (bp) and the G + C content was 69.34%, while the number of rRNAs operons, tRNAs and protein-coding genes were 6, 40, and 3541, respectively. On the other hand, the size of chromosome 2 was 3,115,829 bp, and the G + C content was 69.52%, while the number of rRNAs operons, tRNAs and protein-coding genes were 3, 11, and 2808, respectively. The plasmid A was 129,637 bp in size with the G + C content of 65.83%, while the plasmid B was 32,782 bp in size with the G + C content of 59.62%. The whole genome of this bacterium was 7,110,107 bp in size. Interestingly, the bacteriochlorophyll biosynthesis, light-harvesting proteins and the photosynthetic reaction center related genes were encoded on the chromosome 2, suggesting that the *A. tertiaricarbonis* RN12 strain might have originally been of the photosynthetic ability by itself, which was lost during bacterial evolution and adaptation to specific habitats as consequence of spontaneous mutations and/or transposon/insertion sequence insertional inactivation of certain photosynthesis related genes. Either chlorophyll synthesis or photosynthesis was not observed under light cultivation. It may be that the aerobic anoxygenic photosynthesis (AAP) was not occurred under the energy excess conditions [11]. Similarly, a large exopolysaccharide biosynthesis and secretion gene cluster, similar to that of another floc-forming strain *Zoogloea resiniphila* MMB [2], had been found in the genome of *A. tertiaricarbonis* RN12.

**Table 1:** General characteristics of the *Aquincola tertiaricarbonis* RN12 strain genome

	chromosome 1	chromosome 2	plasmid A	plasmid B
Genome size (bp)	3,831,859	5,141,032	129,637	32,782
GC content (%)	69.34	69.52	65.83	59.62
Number of rRNAs	6	3	0	0
Number of tRNAs	40	11	0	0
Number of protein coding genes	3541	2808	127	30

### Identification of the genes required for floc formation

**Fig. 2:**
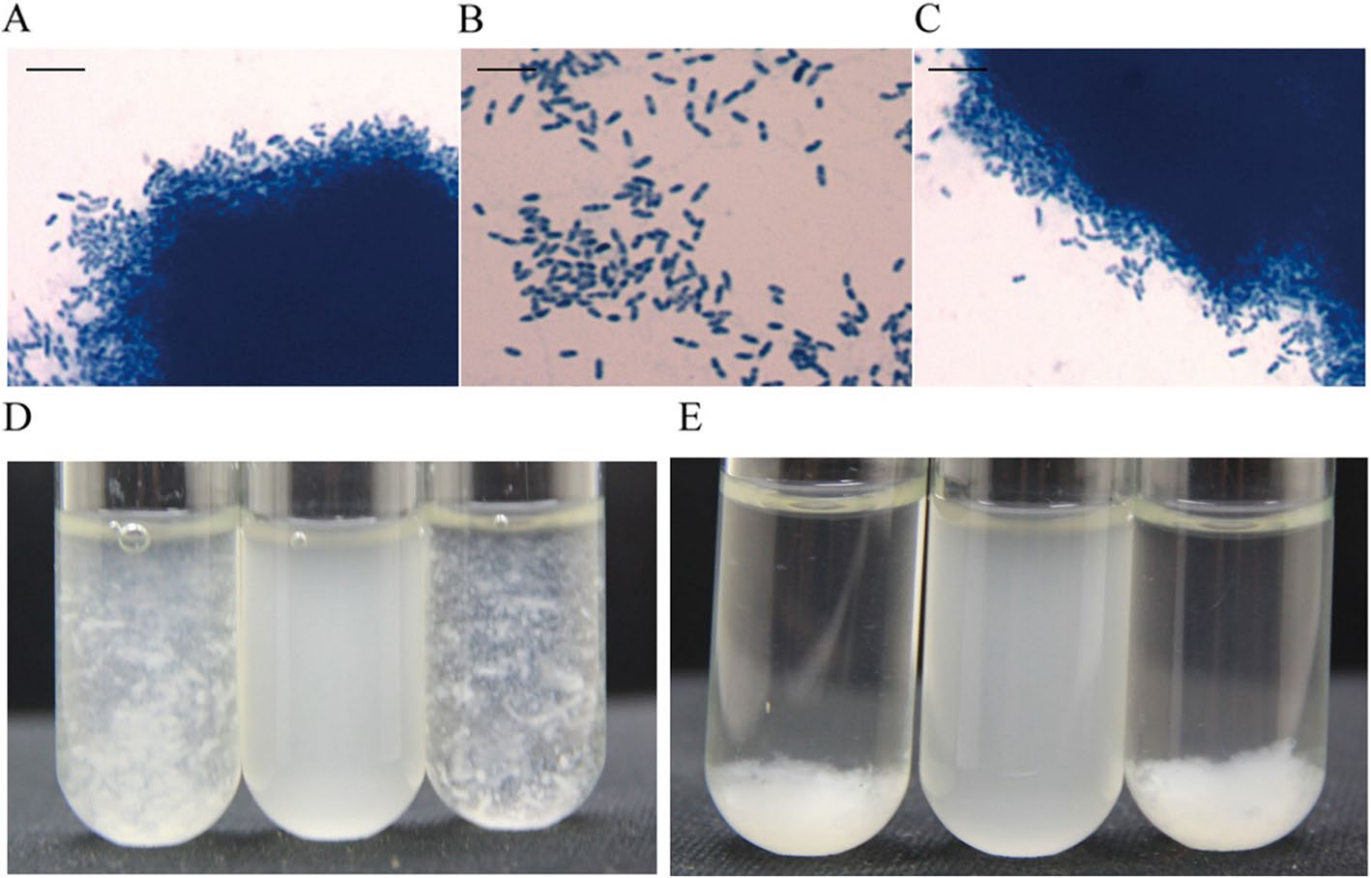
Genetic complementation analyses of the *prsT* disruptant RN12M51 of *A. tertiaricarbonis*. **A** Wild type RN12 strain with pBBR1MCS-5 empty vector visualized by light microscope after staining with methylene blue. **B** Floc-formation deficient mutant RN12M51 with pBBR1MCS-5. **C** Floc-forming RN12M51 complemented by pBBR1MCS-5-*prsT* construct. **D** Agitated bacterial cultures from panels A, B and C, from left to right. **E** Settled bacterial cultures from panels A, B and C, from left to right. **A** to **C** were visualized by light microscope after staining with methylene blue (× 1000 magnification); Bars: 10 μm

**Fig. 3:**
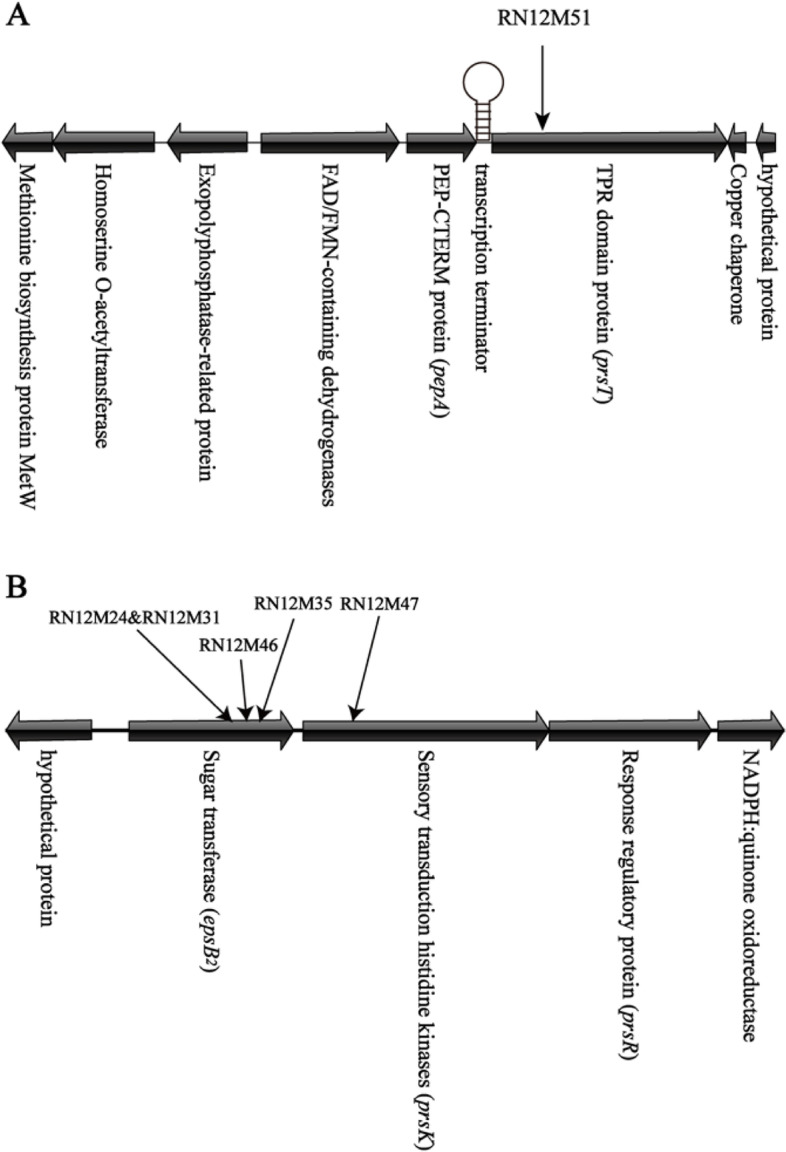
The two gene clusters required for floc formation of *Aquincola tertiaricarbonis* RN12 strain. **A** Organization of the chromosomal region adjacent to the TPR lipoprotein encoding *prsT* gene which was disrupted by transposon insertion (black arrow) mapped in the floc-deficient transposon mutant M51. **B** Organization of the chromosomal region adjacent to the sugar transferase encoding *epsB2* gene and a sensory transduction histidine kinase gene *prsK* which was disrupted by insertion (black arrows) of a *mariner* transposon mapped in the transposon mutants deficient in bacterial floc formation

Large scale transposon mutagenesis was conducted on the wild type *A. tertiaricarbonis* RN12 strain by using the *mariner* transposon delivery vector pmini*Himar* RB1 as previously described [2, 12, 13]. A series of floc-formation-deficient mutants, whose liquid cultures were turbid and planktonic rather than flocculated as observed in the cultures of the wild type RN12 strain, had been isolated and the transposon insertion site was mapped in most of the floc-deficient mutants. The homogenous turbid cell cultures composed of planktonic single cells were visualized by light microscope. In addition to the previously described exopolysaccharide biosynthesis gene cluster [3], two other loci had been identified to be required for floc formation in our study. An experimentally uncharacterized gene encodes a PEP-CTERM sorting domain protein (TIGR02595, designated *pepA* hereafter) and was located upstream of the mapped *prsT* gene (Figs. 2 and 3A), which encodes a putative PEP-CTERM system TPR-repeat lipoprotein (TIGR02917).

Another mapped locus encodes a glycosyltransferase gene designated *epsB2* (TIGR03013), which is a PEP-CTERM system associated sugar transferase and may be a cytoplasmic membrane bound undecaprenyl-phosphate galactose phosphotransferase (Supplemental Fig. S1), and a Fis-type two-component system termed PrsK (TIGR02916, putative PEP-CTERM regulatory system histidine kinase) and PrsR (TIGR02915, PEP-CTERM-box response regulator transcription factor) (Fig. 3B). The EpsB2 protein is a homolog of the EpsB glycosyltransferase found in *Methylobacillus* sp. strain 12S, which is also associated with a PEP-CTERM system, but of a distinct type [10, 14]. Interestingly, these proteins are part of the computationally predicted PEP-CTERM/exosortase system, analogous to the LPXTG/sortase system common in Gram-positive bacteria [10]. The transposon insertion had been mapped to the *epsB2* gene in multiple floc-deficient mutants (Supplemental Table. S2), suggesting its essential role in exopolysaccharide biosynthesis and floc formation. Its downstream gene is *prsK*, encoding sensory transduction histidine kinases, whose gene disrupted mutant is RN12M47 (Fig. 3B). The cellular role of the *prsT* and *epsB2* genes was further confirmed by the genetic complementation analyses in which the plasmid borne *prsT* (Fig. 2) or *epsB2* (Supplemental Fig. S2) gene could restore the floc-forming phenotype to the specific mutant. Previously we also identified a large gene cluster required for the floc formation of RN12 and the activated sludge bacterium *Zoogloea resiniphila* MMB, respectively [2, 3]. However, the plasmid borne *prsK* or *prsK-prsR* cassette failed to recover the floc-forming phenotype to the RN12M47 mutant due to an unknown reason (Supplemental Fig. S3). The predicted orthologues for the genes of these two gene clusters identified in the *A. tertiaricarbonis* RN12 strain are also present in the closely related proteobacterial genomes of *Rubrivivax gelatinosus* IL144 and *Leptothrix cholodnii* SP-6 (Supplemental Table S3).

### Transcription of *pepA* was dependent on RpoN1 (sigma^54^)

**Fig. 4:**
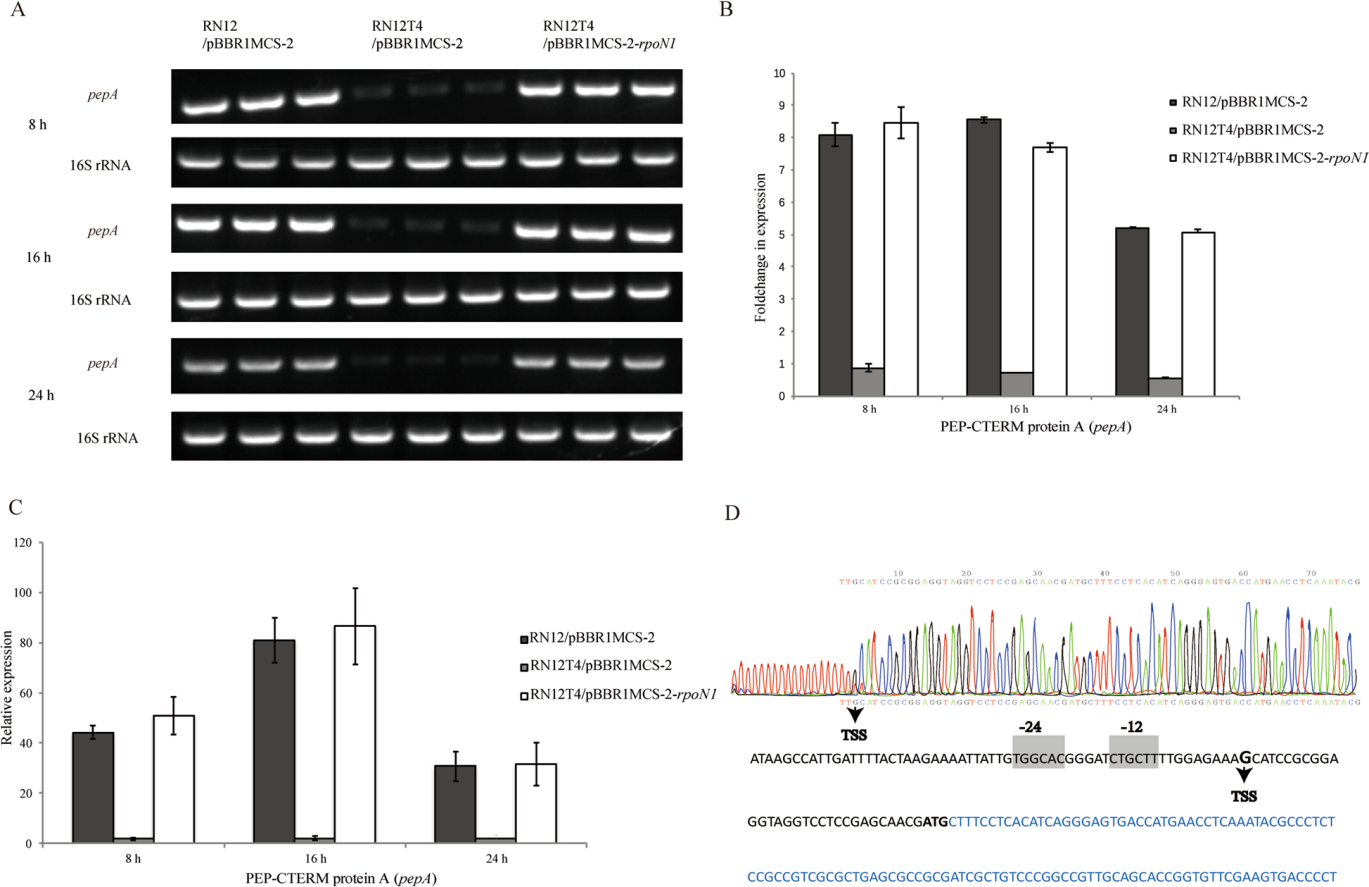
Transciptional analyses showing that the PEP-CTERM gene *pepA* was regulated by the RpoN1 sigma factor. Bacteria were cultivated at 28 °C in the R2A medium with shaking (200 rpm). The data represent the samples collected at 8 h, 16 h and 24 h. **A** Transcription of the *pepA* gene was examined by semi-quantitative RT-PCR with 16S rRNA gene as the loading control. **B** Trace quantity plotting using ‘Quantity One’ software. The experiments were performed in triplicate. **C** Transcription of the gene was examined by real-time PCR, relative transcript levels were calculated according to the 2^−ΔΔCt^ method, 16S rRNA was analyzed and used as an internal control gene. The experiments were performed in triplicate. Error bars represent the standard deviation. **D** Determination of the transcriptional start site (TSS) of the *pepA* gene in *A. tertiaricarbonis* RN12 strain. The blue sequence is the open reading frame of *pepA* and the arrow-pointed nucleotide G is the determined transcriptional start site (+ 1). The shadowed sequences TGGCAC and CTGCTT are the putative − 24 and − 12 motifs of the promoter recognized by the sigma factor RpoN1

An obvious intergenic region (nearly 85 nt) could be found upstream of the open reading frame (ORF) of *pepA*, indicating that there might be complex gene transcriptional regulatory motifs. There is also a putative “stem-and-loop” transcriptional terminator between the ORF of *pepA* and the downstream *prsT* gene (Fig. 3A). The transcriptional start site of *pepA* was determined by a primer extension analysis, demonstrating that the conserved − 24/− 12 motifs GG and GC were present upstream of its ORF (Fig. 4D). Furthermore, the transcription of *pepA* was compared in the wild type RN12 strain (carrying pBBR1MCS-2 empty vector), the *rpoN1* mutant RN12T4 (carrying pBBR1MCS-2) and the complementation strain carrying the pBBR1MCS-2-*rpoN1* construct by using real-time PCR (Fig. 4C) and RT-PCR (Figs. 4A and B)*.* It was clearly demonstrated that high levels of transcription of *pepA* were dependent on the presence of a wild type *rpoN1* gene, which was either present on the chromosome of the wild type strain or encoded in the plasmid in the RN12T4 mutant (*p* < 0.001). On the other hand, the transcription of downstream *prsT* gene was lower and seemed to be not affected by the disruption of *rpoN1* (*p* < 0.001) (Supplemental Fig. S4). These results are consistent with previous bioinformatics prediction that certain PEP-CTERM genes are dependent on RpoN sigma factor and probably the accessory PrsK-PrsR two-component system as well [10]. A previously predicted enhancer sequence could also be found upstream of this preliminarily mapped RpoN-recognized promoter. More experiments are needed to clarify the regulatory mechanism underlying the expression of PEP-CTERM genes.

### Overexpression of *pepA* rescued floc formation by decreasing soluble exopolysaccharides in the *rpoN1*-disupted mutant

**Fig. 5:**
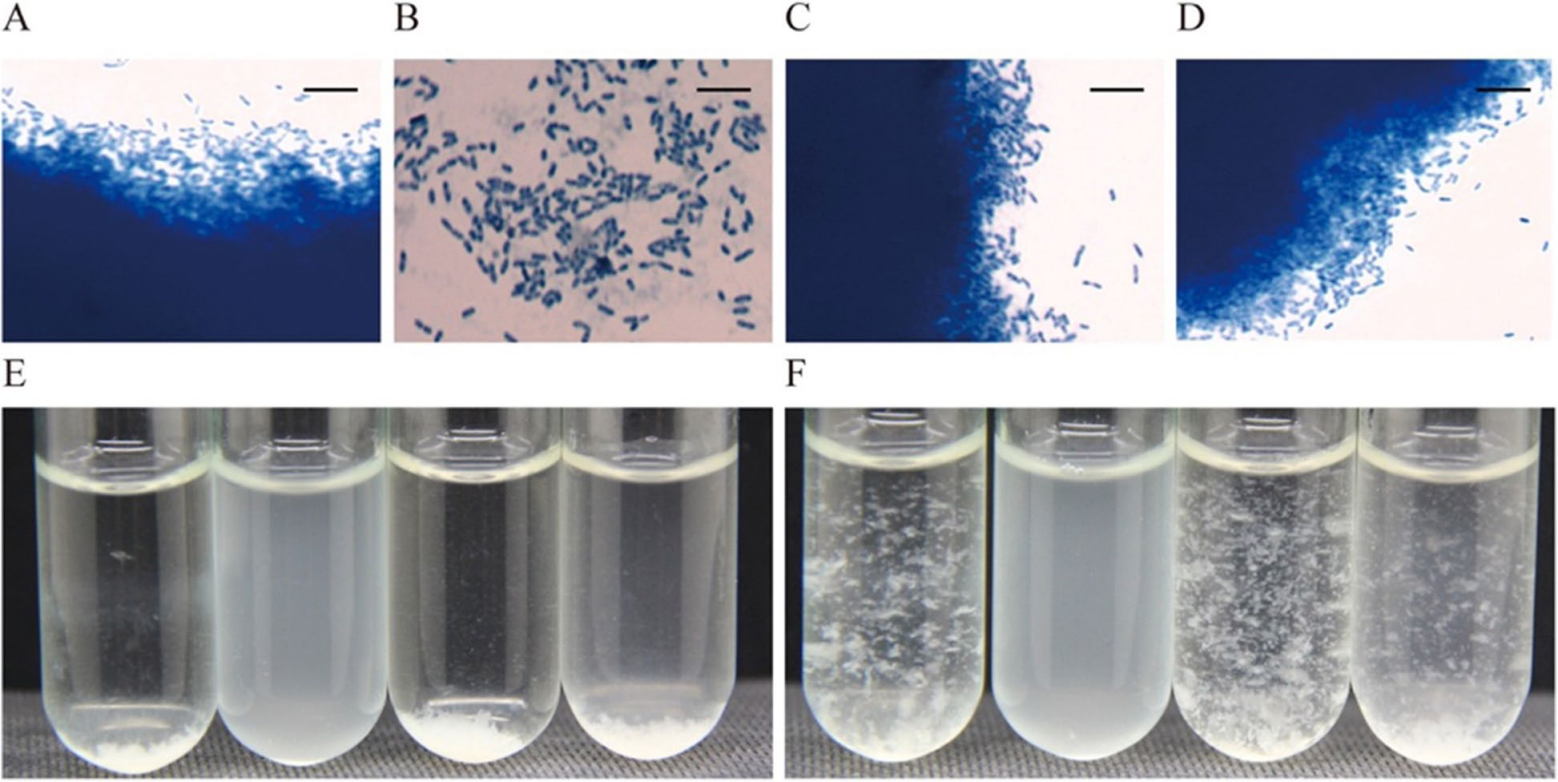
Overexpression of the PEP-CTERM gene *pepA* circumvented the requirement of RpoN1 for floc formation in the RN12T4 mutant. Plasmid borne wild type *pepA* gene recovered the floc-forming phenotype of the RN12T4 mutant similar to that of the wild type strain RN12 with the empty vector. **A** Wild type RN12 strain carrying the pBBR1MCS-2 empty vector. **B** RN12T4 mutant with pBBR1MCS-2 empty vector. **C** RN12T4 mutant with pBBR1MCS-2-*rpoN1* construct. **D** RN12T4 mutant with pBBR1MCS-2-*pepA* construct. **E** Agitated bacterial cultures from panels A to D, from left to right. **F** Settled bacterial cultures from panels (**A**) to (**D**), from left to right. **A** to **D** were visualized by light microscope after staining with methylene blue (× 1000 magnification); Bars: 10 μm

We wondered if overexpression of the PEP-CTERM genes such as *pepA* could rescue the floc-forming phenotype of the *rpoN1*-disruped mutant RN12T4 (Fig. 5). *The PepA PEP-CTERM protein is probably secreted into periplasm* via *Type II secretion system and may be further sorted and translocated out of cells and become part of extracellular polymeric substances required for floc formation.* The so called PEP-CTERM/exosortase system, found in many Gram-negative bacteria including the floc-forming *Aquincola* and *Zoogloea* strains, seems to be involved in the production of the extracellular polymeric substance composed of exopolysaccharides and mature PEP-CTERM proteins [10].

**Fig. 6:**
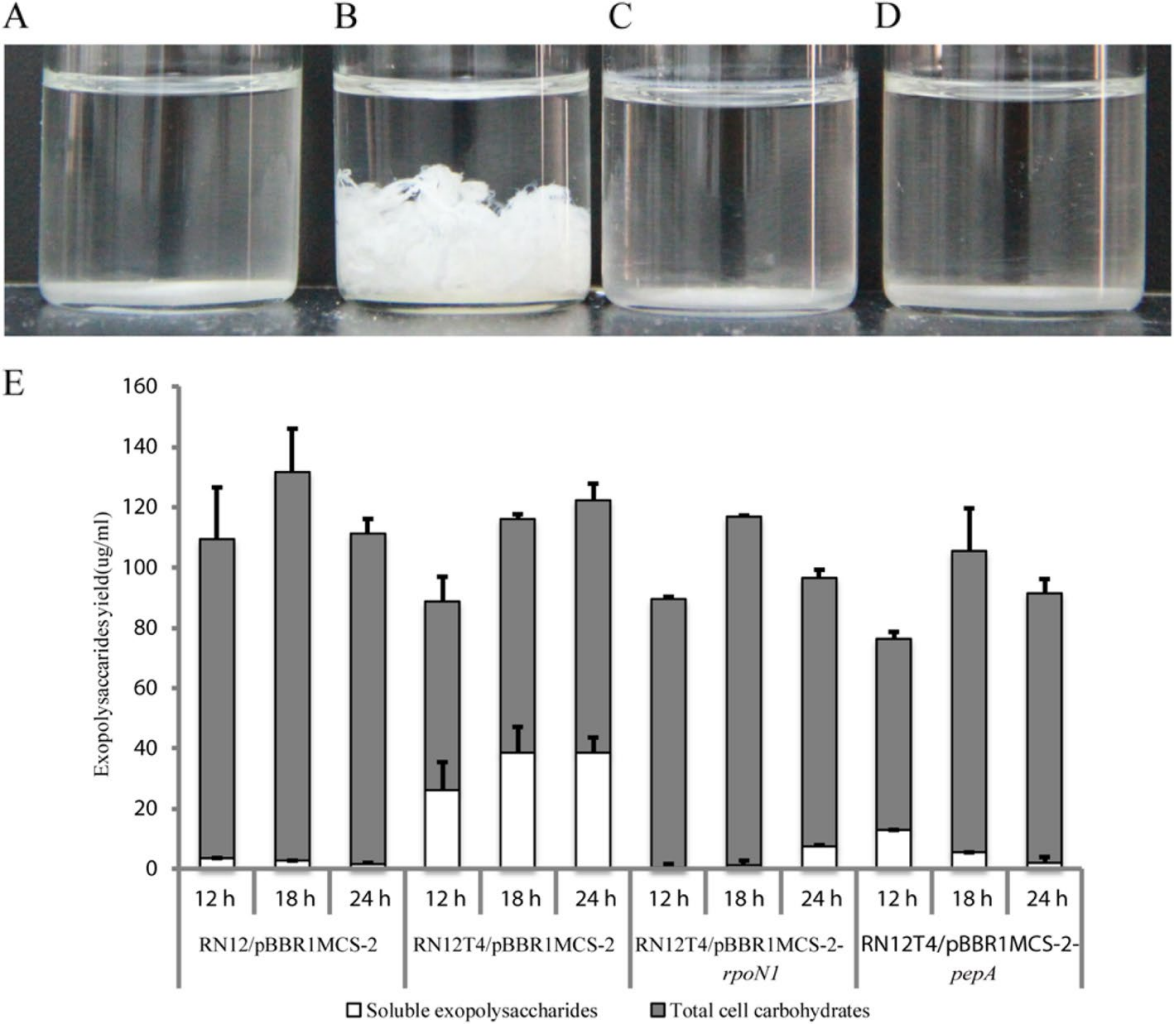
Qualitative and quantitative analyses of the extracellular polysaccharides of *A. tertiaricarbonis* wild type strain and mutants demonstrating that PepA overexpression decreased the release of soluble exopolysaccharides produced by the *rpoN1* mutant. Upper panel: Ethanol-precipitated exopolysaccharides extracted from the cell free supernatants of bacterial cultures of wild type strain RN12 with the pBBR1MCS-2 empty vector (**A**), RN12T4 mutant with the empty vector (**B**), RN12T4 mutant with pBBR1MCS-2-*rpoN1* (**C**) and RN12T4 mutant with pBBR1MCS-2-*pepA* (**D**). Lower panel: Average yields of the total cell carbohydrates (grey bars) and released soluble exopolysaccharides (S-EPS, white bars) levels of bacterial cultures of A, B, C and D were measured using the phenol-sulfuric acid method at 12 h, 18 h and 24 h (**E**). Values were shown as micrograms per milliliter of bacterial suspension. All experiments were performed in triplicate; Error bars represent the standard deviation

Previously it had been revealed that most of the secreted polysaccharides were released and dissolved in the culture broth rather flocculating the bacterial cells when *rpoN1* gene was inactivated [3]. Fiber-like exopolysaccharides could no longer be precipitated and extracted from the cell-free supernatants of bacterial cultures of RN12T4 mutant when the *pepA* gene was overexpressed (Figs. 6A, B, C, and D). Quantitative measurements also demonstrated that the bound exopolysaccharides increased, whereas the soluble exopolysaccharides decreased markedly when the floc-forming phenotype was rescued by the plasmid borne *pepA* gene (*p* < 0.001) (Fig. 6E). These results suggest that the released exopolysaccharides dissolved in the supernatants of culture broth decreased markedly and could no longer be extracted when PepA protein was overexpressed. In the meantime, the bound exopolysaccharides increased and floc-forming phenotype was recovered in the RN12T4 mutant. These facts indicate that *the* exopolysaccharides became to be tightly bound to bulks of bacterial cells to form the flocs via a PEP-CTERM protein mediated process. The secreted PEP-CTERM proteins may interact with extracellular polysaccharide chains to form bound EPS via glycosylation [10], although the underlying mechanism remains to be further investigated.

Interestingly, there are more than 60 PEP-CTERM domain protein encoding genes identified in the genome of *A. tertiaricarbonis* RN12. We chose and tested several other genes, encoding the typical PEP-CTERM domain protein which harbors both N-terminal secreation signal and C-terminal PEP-CTERM domain as well as a high percentage of asparagine residues and nontypical ones. Results show that only the *pepA* gene have the ability to rescue the foc-forming of RN12T4 strain (Supplemental Fig. S5). We suppose that the floc forming of *A. tertiaricarbonis* RN12 require many proteins (including PEP-CTERM domain proteins and some other proteins), and this trait was likely to be maintained or partially maintained just in the absence of *pepA* gene. However, our criterion was absolutely no microbial suspensions in liquids in the process of screening mutants, so no such gene was found. Plasmid borne *pepA* can rescue as a result of the over expression.

### Western blot analysis on the recombinant PepA protein

**Fig. 7:**
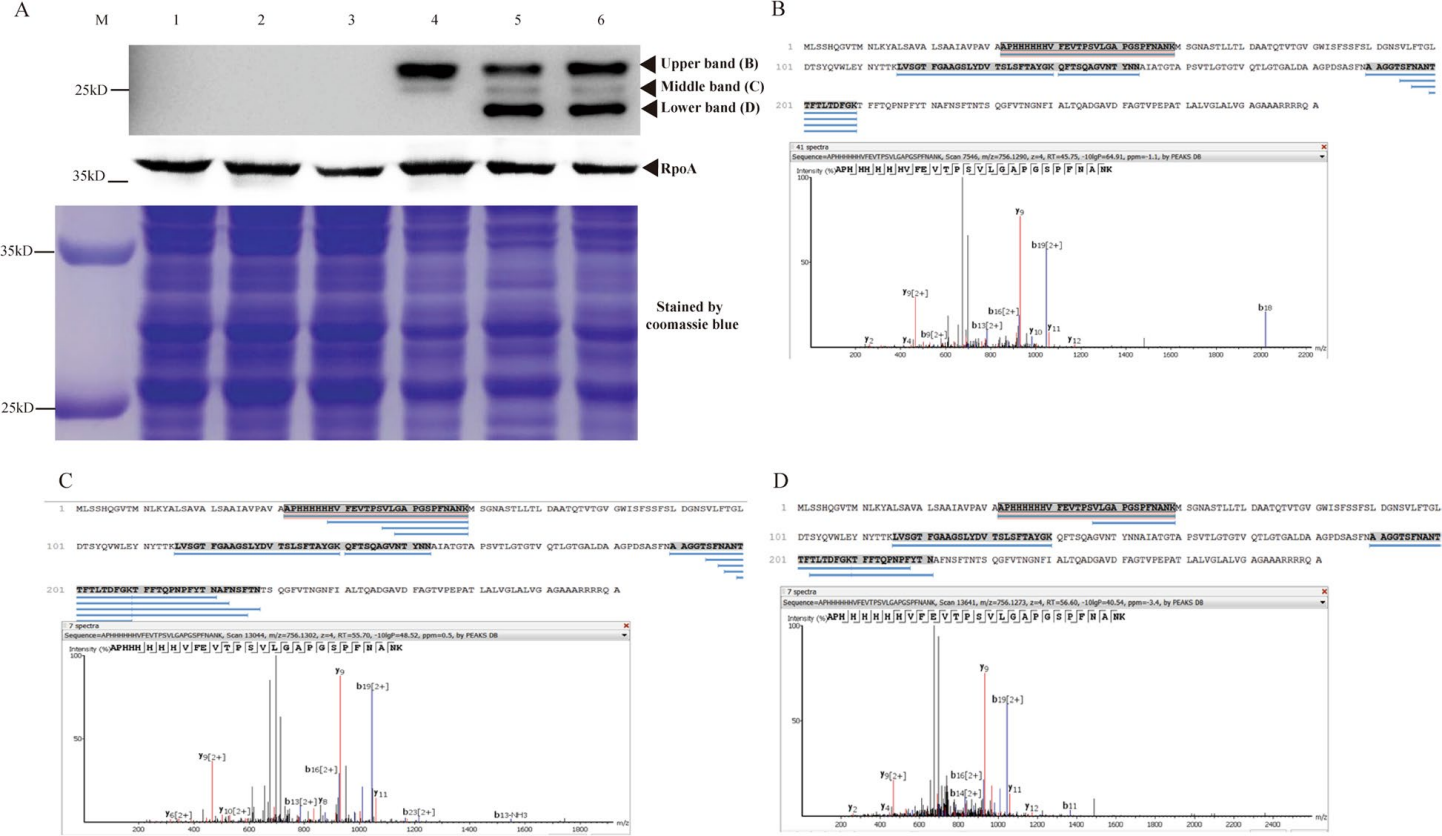
Western blot and tandem mass spectrometry (MS/MS) analyses of His-tagged recombinant PEP-CTERM protein of PepA in the *A. tertiaricarbonis* strains. The samples were taken at 12 h, 18 h and 24 h. Immunoblotting was performed with His-specific monoclonal mouse antibody followed by a goat anti-mouse IgG conjugated to HRP. **A** PVDF membrane incubated with His-tag-specific antibody Western ECL (Upper panel) and three bands, upper, middle and lower, appeared; a PVDF membrane incubated with anti-RpoA antibody (Generated using the RpoA recombinant protein of closely related *Zoogloea resiniphila* MMB strain as the antigen) Western ECL as a loading control (Middle panel) and also a Coomassie Brilliant Blue stain of the SDS-PAGE gel as a loading control (Lower panel). Lane M: The size marker; Lane1-Lane 3: The proteins extracted from the RN12T4 mutant carrying the empty vector pBBR1MCS-2 over the time course; Lane 4-Lane 6: The proteins extracted from RN12T4 mutant carrying pBBR1MCS-2-*pepA* over the time course. The molecular weight of the marker bands is indicated in kilo-Daltons (KD). Mass spectrometry identification of the upper (**B**), middle band (**C**) and lower band (D) detected by the anti-His tag antibody in the Western blots. The MS/MS sequence coverage of proteins is 31, 37 and 30%, respectively. MS/MS spectra of the tryptic peptide APHHHHHHVFEVTPSVLGAPGSPFNANK (*m/z* = 756.129) confirmed the presence of the hexahistindine tag in all three bands. The western blots used in figures were from two regions of one membrane (The RpoA was about 35KD and the PepA was lower than 29KD)

The PEP-CTERM sorting domain proteins usually contain an amino terminal (N-terminal) signal peptide (Supplemental Fig. S6A) and therefore these putative proteins may be secreted into the periplasmic space by the Type II secretion system [10]. The function of the PepA signal peptide was further confirmed by phosphatase A-(PhoA) fusion assay (Supplemental Fig. S6B). To monitor the expression of the recombinant PepA protein in the *A. tertiarcarbonis* mutants, we inserted a polyhistidine (His) tag between the P33 and V34 residues after the intact signal peptide by using DNA recombination technology. This construct still could rescue the floc formation phenotype of RN12T4 mutant, indicating that the His-tag did not affect the cellular function of the recombinant PepA protein. Three bands were detected in the Western blot analyses using anti-His-tag monoclonal antibodies (Fig. 7A). The Histidine-tagged recombinant proteins were purified and subjected them to the UPLC coupled LTQ-Orbitrap Elite ETD Mass Spectrometry analyses. All three bands contains the polyhistindine sequence tag and the downstream PepA peptide sequences, indicating the presence of the recombinant PepA proteins in the targeted bands and a post-translational processing and modification of such a PEP-CTERM protein (Fig. 7B, C and D). The N-terminal signal peptide of the PEP-CTERM protein PepA seemed to be cleaved as computationally predicted while the C-terminus might also be further cleaved, which needs to be further investigated and the role of the VPEP motif also needs to be defined.

### Overexpression of *pepA* did not rescue biofilm formation and swarming ability

**Fig. 8:**
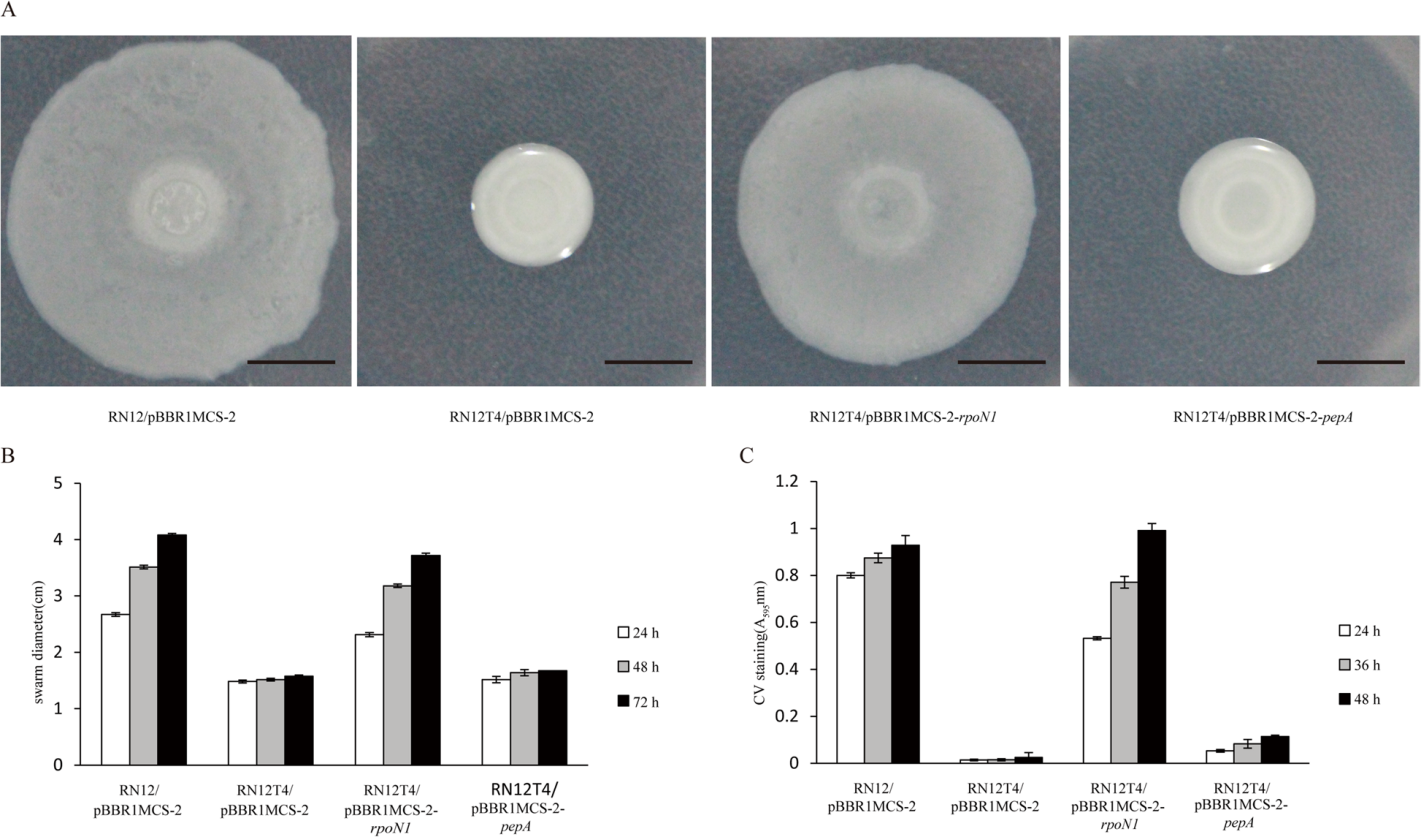
The overexpression of PEP-CTERM gene *pepA* did not rescue the swarming motility and biofilm formation of the *rpoN1* disruptant of *A. tertiaricarbonis.***A** Colony mophology of each strain after three day incubation. Scale bar: 1 cm; **B** Swarming motility expressed as colony diameters at 24 h, 48 h and 72 h; **C** Biofilm levels measured after 24 h, 36 h and 48 h incubation at 28 °C in the R2A media. Each sample was plated in triplicate. Biofilms were stained with 0.1% crystal violet (CV). CV was dissolved with 30% acetic acid and quantified by measuring the optical density at 595 nm; Error bars represent the standard deviation

RpoN sigma factor is a global regulator [15–18] and RpoN1 regulates a series of cellular functions including floc formation, swarming motility and biofilm formation in *A. tertaricarbonis* RN12 [3]. The swarming motility and biofilm formation were monitored in various strains to further reveal the cellular role of PEP-CTERM protein PepA. Neither swarming motility nor biofilm formation was rescued by plasmid-mediated overexpressing *pepA* gene in the *rpoN1* mutant RN12T4 (Fig. 8). It is suggested that *pepA* and/or other PEP-CTERM genes are only involved in floc formation but not biofilm formation or swarming motility.

Taken together, our results demonstrated that the RpoN1-regulated *pepA* gene and probably other PEP-CTERM genes were involved in the floc formation of *A. tertiaricarbonis* RN12 strain. Furthermore, our results also indicated that EPS components involved in the formation of flocs, the microbial suspensions in liquids, and the formation of biofilm, attached to surfaces, seemed to be different, despite similar extracellular polymeric matrices participated in the formation of these two important bacterial aggregates. Further structural analyses on the extracellular polymeric substances, including exopolysaccharides and PEP-CTERM proteins, are needed to reveal the mechanisms underlying the floc formation of *Aquincola, Zoogloea* and other bacteria.

## Conclusions

In this study we have identified several genes involved in floc formation of *Aquincola tertiaricarbonis* RN12 strain, in addition to the previously identified large gene cluster for exopolysaccharide biosynthesis and an RpoN1 sigma factor. The RpoN1 sigma factor regulates the transcription of a PEP-CTERM gene, whose overexpression could prevent the release of exopolysaccharides and rescue the floc formation of the *rpoN1* mutant. These results indicate that floc formation is a tightly regulated process and the RpoN1-dependent PEP-CTERM proteins are involved in self-flocculation of bacterial cells of *Aquincola* and probably other bacteria.

## Methods

### Bacterial strains, plasmids, and culture conditions

The bacterial strains and plasmids used in this study are listed in Table 2. Bacterial strains were cultured in the Luria-Bertani broth (5 g/L yeast extract, 10 g/L tryptone, 10 g/L NaCl, pH 7.0), R2A medium [19], and *Zoogloea* medium (ZM) [19] (supplemented with 15 μg/ml of gentamycin or 50 μg/ml of kanamycin, 50 μg/ml of diaminopimelic acid, and 1.5 g/L Biosharp agar when necessary). The *A. tertiaricarbonis* RN12 strains were grown at 28 °C.

**Table 2:** Bacterial strains and plasmids used in this study

Strains or plasmids	Description	Source or reference
Strains		
*E. coli* DH5α	*F*^*−*^*endA1 glnV44 thi-1 recA1 relA1 gyrA96 deoRnupG Φ80dlacZΔM15 Δ(lacZYA-argF)U169, hsdR17(r*_*K*_^*−*^*m*_*K*_^*+*^*) phoA λ*^*−*^	Takara
*Escherichia coli* WM3064	*thrB1004 pro thirpsLhsdS lacZDM15 RP4–1360 (araBAD)567dapA1341::[ermpir(wt)]*	W. Metcalf
*E. coli* EC100D^+^	*F*^*−*^*mcrA Δ(mrr-hsdRMS-mcrBC) φ80dlacZΔM15 ΔlacX74 recA1 endA1 araD139 Δ(ara, leu)7697 galUgalK λ*^*−*^*rpsL (Str*^*R*^*) nupGpir*^*+*^*(DHFR)*	Epicentre Technologies
RN12WT	Floc-forming strain	(3)
RN12T4 mutant	mariner transposon insertion in *rpoN1* at nt 1274	(3)
RN12M35 mutant	mariner transposon insertion in *epsB2* at nt 1295	This study
RN12M51 mutant	mariner transposon insertion in *prsT* at nt 2097	This study
plasmids		
pminiHmarRB1	The mariner transposon delivery plasmid R6K	(15)
pBBR1MCS-2	Broad-host-range shuttle vector, Km^r^	(20)
pBBR1MCS-5	Broad-host-range cloning vector, Gm^r^	(20)
pBBR1MCS2-*rpoN1*	*rpoN1* gene cloned in pBBR1MCS-2, Km^r^	(3)
pBBR1MCS2-*pepA*	*pepA* gene cloned in pBBR1MCS-2, Km	This study
pBBR1MCS-5-*prsT*	*prsT gene* cloned in pBBR1MCS-5, Gm^r^	This study
pBBR1MCS-5-*epsB2*	*epsB2 gene* cloned in pBBR1MCS-5, Gm^r^	This study

### Bioinformatics analysis

Multiple sequence alignments were performed by the Clustal X alignment program [20]. Hidden Markov models (HMMs) previously constructed [10] were used to search for PEP-CTERM proteins. The two-tailed independent-sample t-test was conducted to test the significance level in this manuscript.

### Transposon mutagenesis and genetic complementation

The *mariner* transposon mutant libraries were generated as previously described [12, 21]. *Escherichia coli* WM3064 strain carrying the transposon delivery suicide plasmid, pmini*Hmar* RB1 (courtesy by Dr. Daad Saffarini) as the donor strain and the *A. tertiaricarbonis* RN12 strain as the recipient strain for biparental conjugation. After 4–6 h of mating on LB agar plates supplemented with diaminopimelic acid, the bacterial cells were diluted and plated on R2A agar plates supplemented with kanamycin (50 μg/ml) and the mutants deficient in floc formation were screened out. The transposon insertion site in each mutant was mapped as previously described [22]. The chromosomal DNA of these non-floc forming strains were digested with *Sph*I and selfligated by T4 DNA ligase (Takara, Dalian, China). Then, the circular closed DNA was introduced into the EC100D pir^+^ Electrocompetent *E. coli* through electroporation [23, 24]. Finally, the plasmids were extracted and sequenced to identify the insertion site. For genetic complementation analyses, the target genes were PCR amplified and cloned into the pBBR1MCS-5 vector [25] (primers and restriction sites were shown in Table S1). The recombinants and empty vector were transferred into the *A. tertiaricarbonis RN12* wild type strain and mutant strains via conjugation using WM3064 as the donor strain.

### Exopolysaccharide quantification

Due to the fact that the exopolysaccharides were released into the medium in the RN12T4 mutant, we divided exopolysaccharides of cells into two sections: soluble exopolysaccharides and total cell carbohydrates as previously described [3]. The soluble exopolysaccharides extraction was conducted as previously described [26]. Wild type and mutant strain were grown at 28 °C in R2A broth with shaking (200 rpm). 1.5 ml culture were used to centrifuge for the further analysis of soluble exopolysaccharides and total cell carbohydrates. In the supernatants, soluble exopolysaccharides of strains were precipitated by addition of 3 volumes of ice-cold 95% (v/v) ethanol and then were measured by the phenol-sulfuric acid method using D-glucose as a standard [27]. In the pellets, the total cell carbohydrate contents were directly measured by the phenol-sulfuric acid method using D-glucose as a standard [27]. Concentration of EPS was expressed as micrograms per milliliter of bacterial culture.

### Biofilm and swarming motility assay

Relative biofilm production levels were assayed using the 96-well crystal violet staining method as described previously [28, 29]. Bacteria were cultivated overnight in R2A broth and then were diluted by 20 folds with fresh broth. One hundred μL of the diluted cultures were placed into each 96-well plate. Every sample was plated in triplicate and the wild type strain used as control for each plate. The triplicate plates were grown at 28 °C for 24 h, 36 h and 48 h, respectively. After that, the staining of biofilm was conducted by crystal violet as previously described and the formation of biofilm was monitored by measuring optical density at 595 nm using a Thermomax spectrophotometer [3].

Swarming motility was assayed using soft R2A agar medium with 0.4% agar following the procedure as previously described [30, 31]. Bacteria were cultivated overnight in R2A broth and 5 μL of the culture was plated on the central of an individual petri dish in triplicate. Motility was visualized as a white halo as a result of cells moving outward from the original inoculation site. The diameters of the colonies were measured and photographed after 24 h, 48 h and 72 h of incubation at 28 °C.

### RNA extraction, real-time PCR analysis of gene transcription

Samples were taken for RNA extraction from the RN12/pBBR1MCS-2, RN12T4/pBBR1MCS-2 and RN12T4/pBBR1MCS-2-*rpoN1* after 12 h, 18 h and 24 h. Total RNA was extracted using RNAiso Plus (Takara) and RNAprep pure Cell/Bacteria Kit (TIANGEN BIOTECH (Beijing) CO., LTD.) according to the manufacturer’s instructions followed by DNase I treatment. The total RNA were then purified and cDNA were prepared as previously described [3]. Semi-quantitative PCR analyses were carried out as described previously [32]. Quantitative real-time PCR was performed in 20 μL total volume in which 1 μL of 10-fold diluted cDNA was used as the template. The relative gene expression levels were quantified using SYBR Premix DimerEraser (Takara) on a Roche LightCycler 480 II Real-Time PCR system (Roche Diagnostics, Penzberg, Germany). Cycling conditions were as follows: 5 min at 95 °C, followed by 27–30 cycles of 30 s at 95 °C, 30 s at 51–60 °C and 30 s at 72 °C. The gene expression was then normalized against the 16S rRNA gene by using the 2^−ΔΔCt^ method [33]. The expression of each gene was determined by averaging three replicates. The primers used are listed in the Supplemental Table S1.

### Determination of transcription start site

Terminal deoxynucleotidyl transferase (TdT, Takara) was used to incorporate the single deoxynucleotides (dATPs) into the 3′-OH terminus of cDNA to make the dA-tailed cDNA according to the manufacturer’s instructions. Then, touch down and nested PCR were used to amplify the dA-tailed cDNA by using an oligdT (5′-gccagtcTTTTTTTTTTTTTTTTT-3′) primer and a gene-specific primer [32, 34]. The PCR product was cloned into pMD18-T vector (Takara, Dalian, China) for sequencing.

### SDS-PAGE electrophoresis and Western blot analysis

Bacterial cultures were grown in R2A broth with 15 μg/ml of gentamycin and/or 50 μg/ml of kanamycin at 28 °C and 200 rpm for 12 h, 18 h and 24 h. The harvested cells were diluted with sample buffer and then homogenized by using Ultrasonic Cell Disruption System (SCIENTZ-IID, Ningbo Xingzhi Biotechnology Co., China) and centrifuged at 4 °C. The supernatants containing the cellular protein fraction were mixed with the SDS loading buffer and boiled for 10 min followed by electrophoresed by 12% SDS-PAGE. After electrophoresis, gels were electroblotted to polyvinylidene difluoride membrane in transfer buffer (47.8 mM Tris, 36.7 mM glycine, 1.3 mM SDS, 20% methanol) and the blotted membrane was blocked in TBS/5% skim milk powder for 2 h. For immunodetection，His-tagged proteins were probed with His-specific monoclonal primary antibodies (Beyotime) at 1:1000 dilution overnight. Immumoblots were rinsed three times with TBS/0.1% Tween 20 followed by one time with TBS and immuno-coupled for 1 h with anti-Mouse IgG (H + L)-HRP (Beyotime) according to manufacturer’s instructions. Three washing steps with TBS/0.1% Tween 20 of 10 min were followed by 10 min of incubation in TBS. After this step, ECL Plus (Biosharp) was used for detection and film images were digitized using ImageQuant LAS4000mini (Japan). An antiserum generated by using the recombinant protein of alpha subunit of RNA polymerase (RpoA) of *Zoogloea resiniphila* MMB strain in the rabbit was used for a loading control [2].

### Tandem mass spectrometry analyses of his-tagged proteins

Three coomassie blue stained SDS-PAGE gel bands corresponding to those targeted by the anti-His-tag antibody in the immunoblots were excised and destained in 25 mM ammonium bicarbonate-50% acetonitrile at least 3 times and dehydrated with acetonitrile, then reduced with 25 mM DTT in 50 mM ammonium bicarbonate at 56 °C for 25 min, and alkylated with 55 mM iodoacetamide at room temperature in the dark for 30 min. After washing and dehydration in 25 mM ammonium bicarbonate-50% acetonitrile and absolute acetonitrile, gel pieces were digested with 10 ng/ μL mass spectrometry grade trypsin gold (Promega, V5280) or chymotrypsin (Promega, V1062) in 25 mM ammonium bicarbonate at 37 °C for 16 hrs. Peptides were extracted sequentially with 20 mM ammonium bicarbonate, 50% acetonitrile and 5% formic acid, and then pooled peptides were evaporated to dryness in acid-resistant CentriVap centrifugal vacuum concentrator (Labconco Kansas City, MO), and re-suspended with 20 μL of 2% methonal-1% formic acid. NanoLC-MSMS analysis of in-gel digested peptide was performed on Waters® nanoACQUITY UPLC® coupled LTQ-Orbitrap Elite ETD Mass Spectrometer (Thermo Fisher Scientific) as previously described [35].

## Supplementary Information

**Additional file 1: Table S1.** Primers used in this study. **Table S2.** Identification of the insertional sites of transposon mutants of *Aquincola tertiaricarbonis* RN12 defective in the floc formation. **Table S3.** The gene products of the two gene clusters identified in *A. tertiaricarbonis* RN12 strain and the predicted orthologues in the closely related proteobacterial genomes of *Rubrivivax gelatinosus* IL144 and *Leptothrix cholodnii* SP-6 (the polypeptide sequence identity was shown). **Supplemental Fig. S1.** The glycosyltransferase EpsB2 is a putative cytoplasmic membrane bound protein with five transmembrane domains as computationally predicted by using Protter software. Five transmembrane domains have been predicted, but the actual topological traits of this glycosyltransferase remains to be characterized experimentally. **Supplemental Fig. S2.** The genetic analysis of the glycosyl transferase gene *epsB2* in the RN12M35 transposon insertional mutant deficient in floc formation. **Supplemental Fig. S3.** The genetic analysis of the *prsR* gene in the RN12M47 transposon insertional mutant deficient in floc formation. **Supplemental Fig. S4.** Transcriptional analyses of the *prsT* gene downstream of *pepA* showed RpoN1-independent expression. **Supplemental Fig. S5.** The genetic analysis of PEP-CTERM genes in the RN12T4 transposon insertional mutant deficient in floc formation. **Supplemental Fig. S6.** The alkaline phosphatase A (PhoA)-fusion assay demonstrated that the PEP-CTERM protein PepA is secreted into the periplasm as computationally predicted. **Supplemental Fig. S7.** Transcription of the gene was examined by semi-quantitative RT-PCR with 16S rRNA gene as the loading control. **Supplemental Fig. S8.** The full gels and blots.

## Data Availability

The complete genome of *Aquincola tertiaricarbonis* RN12 strain has been submitted in the GenBank under accession number CP097635 (chromosome 1), CP097636 (chromosome 2), CP097637 (plasmid A), and CP097638 (plasmid B).
