# Supplementary material for: An RpoN-dependent PEP-CTERM gene is involved in floc formation of an Aquincola tertiaricarbonis strain
Source: BMC Microbiol. 2023 Jan 19;23:20. doi: 10.1186/s12866-022-02745-1 (PMC9850573; doi:10.1186/s12866-022-02745-1)
Supplement: Supplementary file 1 — Additional file 1: Table S1. Primers used in this study. Table S2. Identification of the insertional sites of transposon mutants of Aquincola tertiaricarbonis RN12 defective in the floc formation. Table S3. The gene products of the two gene clusters identified in A. tertiaricarbonis RN12 strain and the predicted orthologues in the closely related proteobacterial genomes of Rubrivivax gelatinosus IL144 and Leptothrix cholodnii SP-6 (the polypeptide sequence identity was shown). Supplemental Fig. S1. The glycosyltransferase EpsB2 is a putative cytoplasmic membrane bound protein with five transmembrane domains as computationally predicted by using Protter software. Five transmembrane domains have been predicted, but the actual topological traits of this glycosyltransferase remains to be characterized experimentally. Supplemental Fig. S2. The genetic analysis of the glycosyl transferase gene epsB2 in the RN12M35 transposon insertional mutant deficient in floc formation. Supplemental Fig. S3. The genetic analysis of the prsR gene in the RN12M47 transposon insertional mutant deficient in floc formation. Supplemental Fig. S4. Transcriptional analyses of the prsT gene downstream of pepA showed RpoN1-independent expression. Supplemental Fig. S5. The genetic analysis of PEP-CTERM genes in the RN12T4 transposon insertional mutant deficient in floc formation. Supplemental Fig. S6. The alkaline phosphatase A (PhoA)-fusion assay demonstrated that the PEP-CTERM protein PepA is secreted into the periplasm as computationally predicted. Supplemental Fig. S7. Transcription of the gene was examined by semi-quantitative RT-PCR with 16S rRNA gene as the loading control. Supplemental Fig. S8. The full gels and blots. [file 12866_2022_2745_MOESM1_ESM.docx]

**Supplemental Material**

**Table S1** Primers used in this study

| Primer use and name | Sequence(5’-3’) | Restriction site |
| --- | --- | --- |
| Complementation primers | | |
| *prsT*-F | 5’-GGGGTACCCACGGCCCCAGCATCGATTT-3’ | *Kpn*I at 5’ end |
| *prsT*-R | 5’-GCGTCGACTCAGAGTGCTTGAAGCATCC-3’ | *Sal*I at 5’ end |
| *epsB2*-F | 5’-CCAAGCTTTGTGGTTCGGTTATTCAATC-3’ | *Hind*III at 5’ end |
| *epsB2*-R | 5’-GCTCTAGAttagcgcgcgcccttgccgg-3’ | *Xba*I at 5’ end |
| *pepa*-F | 5’-GGAATTCGCCGACATGATGCCAGGAGT-3’ | *Ecor*I at 5’ end |
| *pepa*-R | 5’-GCTCTAGATCGTTGACTTCTGACGCTTCC-3’ | *Xba*I at 5’ end |
| *Pepa*-his F | 5’-CATCATCATCATCATCACGTGTTCGAAGTGACCCCTAG-3’ |  |
| *Pepa*-his R | 5’-GTGATGATGATGATGATGCGGTGCTGCAACGGCCGGGA-3’ |  |
| Real-time PCR primers | | |
| Q-*pepa*-F | 5’-GAACCTCAAATACGCCCTCTC-3’ |  |
| Q-*pepa*-R | 5’-GCATTGCCGCTCATCTTG-3’ |  |
| Q-*prsT* -F | 5’-GTGCTGGACACCCTCGCTAAG-3’ |  |
| Q-*prsT*-F | 5’-ATCCGCTGTGCCTCGCTC-3’ |  |
| RT-PCR primers | | |
| RT- *pepa* -F | 5’-CAACAAGATGAGCGGCAATG-3’ |  |
| RT- *pepa* -R | 5’-TCGGCTGCGTGAAGAAGG-3’ |  |
| RT- *prsT* -F | 5’-CGTCGTGGGCAGTGTTA-3’ |  |
| RT- *prsT* -R | 5’-GCAATGGCTTGGTTCG-3’ |  |
| Primer extension primers | | |
| *pepa* SP1 | 5’-GAACTGTTTGCCGTAG-3’ |  |
| *pepa* SP2 | 5’-GTTGTATTCCAGCCAGACTTG-3’ |  |
| oligo(dT) | 5’-GCCAGTCTTTTTTTTTTTTTTTTT-3’ |  |

**Table S2** Identification of the insertional sites of transposon mutants of *Aquincola tertiaricarbonis* RN12 defective in the floc formation

| Gene | Mutants | Floc formation | Insertion site |
| --- | --- | --- | --- |
| *epsB2* | M24 & M31 | defective | GGAAATTCCGTACTACGCCGTGCG |
|  | M35 | defective | GTACGACCTGTACTACGTGAAGAA |
|  | M46 | defective | CGCAGGTGCGTTACCACTACGGCG |
| *prsT* | M51 | defective | TGATCAGGATCTACACAGCCGAAA |

**Table S3** The gene products of the two gene clusters identified in *A. tertiaricarbonis* RN12 strain and the predicted orthologues in the closely related proteobacterial genomes of *Rubrivivax gelatinosus* IL144 and *Leptothrix cholodnii* SP-6 (the polypeptide sequence identity was shown).

| Gene name | *A. tertiaricarbonis* RN12 protein^a^ | Predicted function^b^ | Polypeptide sequence identity (%) between RN12 and: | |
| --- | --- | --- | --- | --- |
|  |  |  | ***Rubrivivax gelatinosus* IL-144** | ***Leptothrix cholodnii***  **SP-6** |
| *epsB2* | Sugar transferase | EPS unit biosynthesis | 60.2 | 39.3 |
| *prsK* | PEP-CTERM system histidine kinase PrsK | transmembrane histidine kinase | 50.7 | 17.9 |
| *prsR* | PEP-CTERM-box response regulator transcription factor | sigma-54-interacting response regulator | 73.5 | 39.5 |
| MBQ1764872 | NADPH:quinone oxidoreductase | reduces [quinones](https://en.wikipedia.org/wiki/Quinone) to hydroquinones | 52.4 | — |
| KAB2926237 | FAD-binding oxidoreductase | [oxidoreductase activity](https://www.ebi.ac.uk/QuickGO/term/GO:0016614) | — | — |
| *pepA* | PEP-CTERM sorting domain-containing protein | Unknown | 30 | 23 |
| *prsT* | TPR protein | Protein scaffold for EPS synthesis | 39.4 | 13.8 |

^a^Proteins shown in boldface are encoded by those genes disrupted by a transposon insertion. TPR, tetratricopeptide repeat.

^b^EPS, exopolysaccharide.


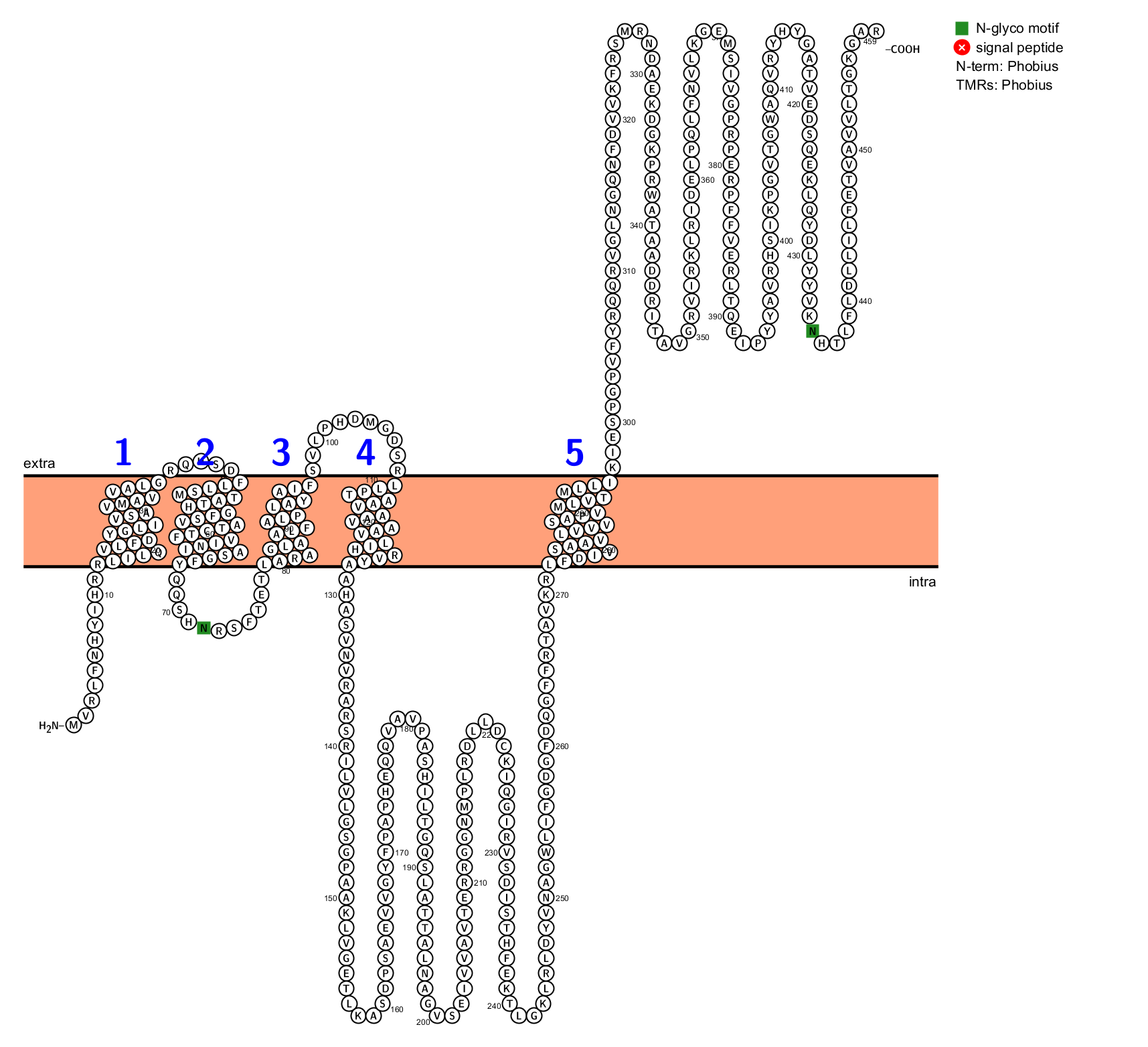


**Supplemental Figure S1** The glycosyltransferase EpsB2 is a putative cytoplasmic membrane bound protein with five transmembrane domains as computationally predicted by using Protter software. Five transmembrane domains have been predicted, but the actual topological traits of this glycosyltransferase remains to be characterized experimentally.

**
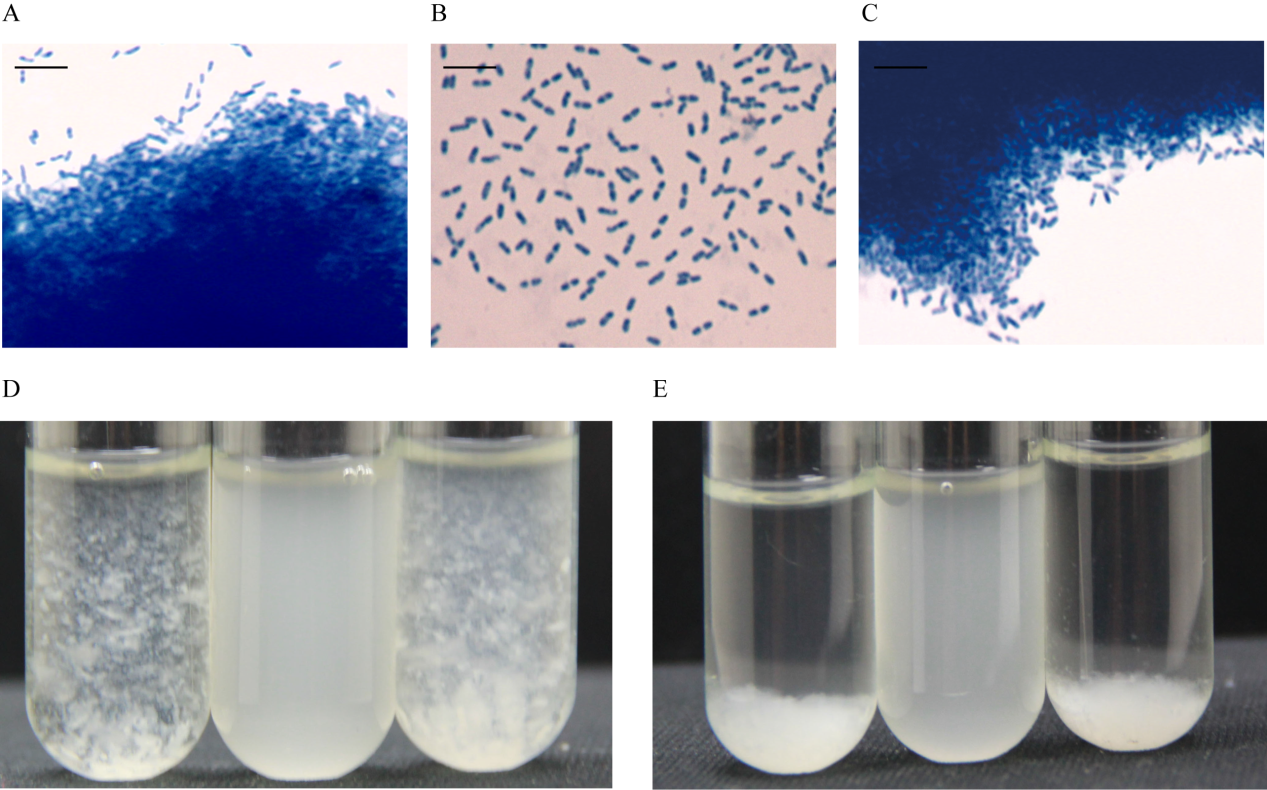
**

**Supplemental Figure S2** The genetic analysis of the glycosyl transferase gene *epsB2* in the RN12M35 transposon insertional mutant deficient in floc formation. (A) Wild-type strain with pBBR1MCS-5 empty vector under microscope. (B) The RN12M35 mutant with pBBR1MCS-5. (C) The RN12M35 mutant with pBBR1MCS-5-*epsB2* construct. (D) Photograph of agitated bacterial cultures and (E) The settled bacterial cultures from panels A to D, from left to right. (A) to (C) were visualized by light microscope after staining with methylene blue (×1000 magnification); Bars: 10 μm.

**
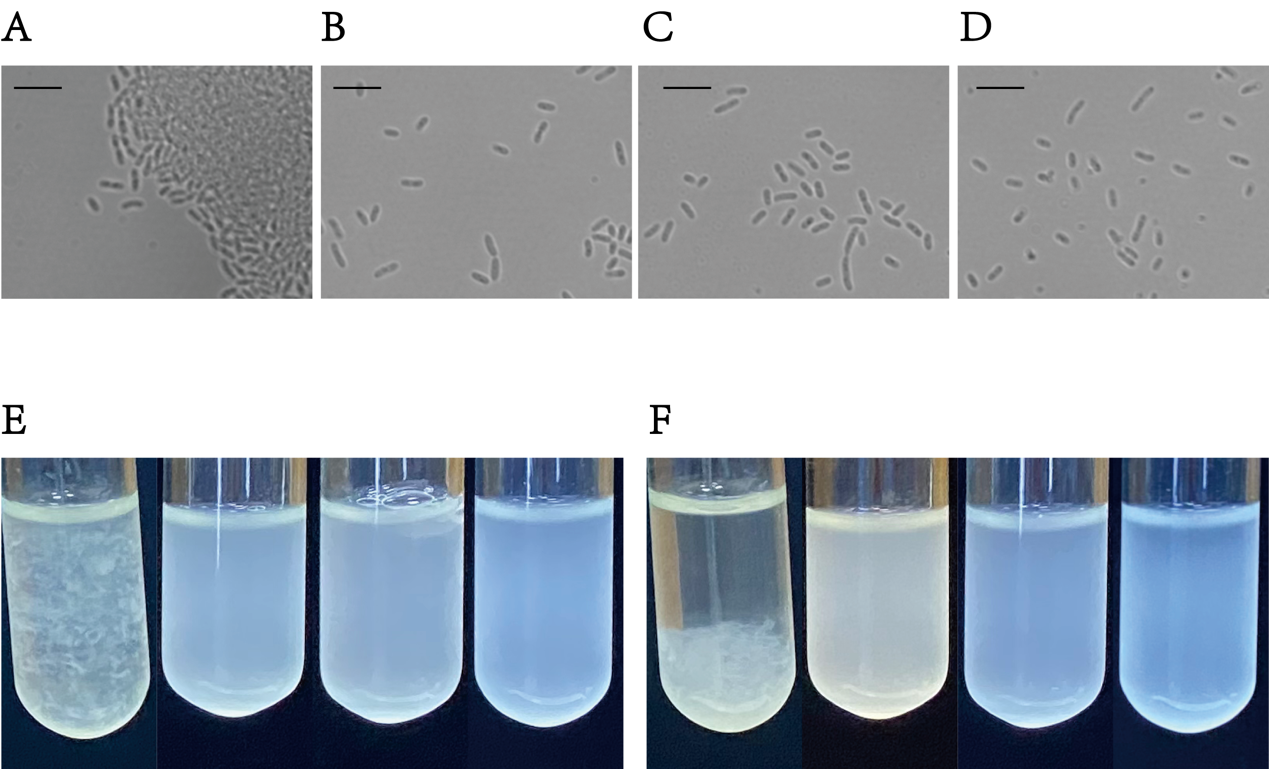
**

**Supplemental Figure S3** The genetic analysis of the *prsR* gene in the RN12M47 transposon insertional mutant deficient in floc formation. (A) Wild-type strain with pBBR1MCS-5 empty vector under microscope. (B) The RN12M47 mutant with pBBR1MCS-5. (C) The RN12M47 mutant with pBBR1MCS-5-*prsR* construct. (D) The RN12M47 mutant with pBBR1MCS-5-*prsR-prsK* construct. (E) Photograph of agitated bacterial cultures and (F) The settled bacterial cultures from panels A to D, from left to right. (A) to (D) were visualized by light microscope (×1000 magnification); Bars: 10 μm.

**
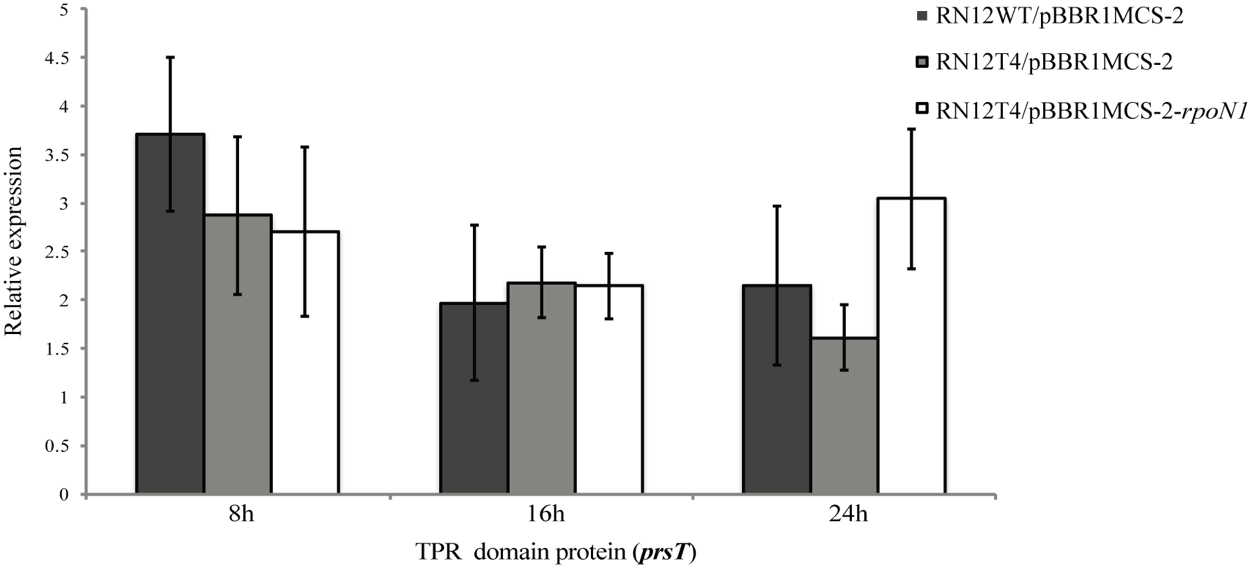
**

**Supplemental Figure S4** Transcriptional analyses of the *prsT* gene downstream of *pepA* showed RpoN1-independent expression. The wild type RN12 strain and the *rpoN1*-disrupted mutant RN12T4 were cultivated at 28°C in the R2A media with shaking (200 rpm). Transcription of the *prsT* gene was examined by real-time PCR, relative expression levels were calculated according to the 2^−ΔΔCt^ method ([1](#_ENREF_1)) with 16S rRNA gene as an internal control gene, and the wild-type RN12 as a calibrator. The assays were performed in triplicates. Error barsrepresent the standard deviation.


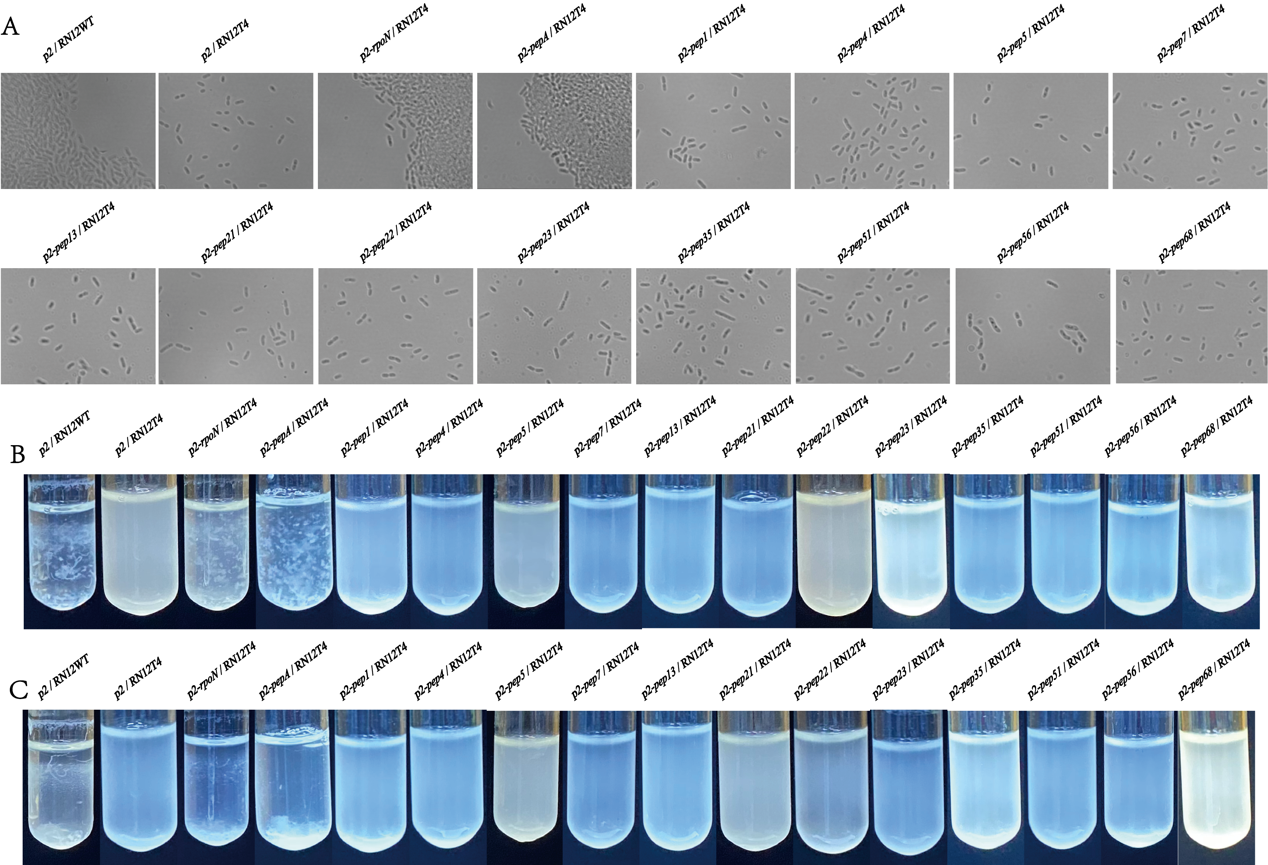


**Supplemental Figure S5** The genetic analysis of PEP-CTERM genes in the RN12T4 transposon insertional mutant deficient in floc formation. (A) The wild-type strain with pBBR1MCS-2 empty vector, the RN12T4 mutant with pBBR1MCS-2, with pBBR1MCS-2-*rpoN*, with pBBR1MCS-2-*pepA*, with pBBR1MCS-2-*pep1*, with pBBR1MCS-2-*pep4*, with pBBR1MCS-2-*pep5*, with pBBR1MCS-2-*pep7*, with pBBR1MCS-2-*pep13*, with pBBR1MCS-2-*pep21*, with pBBR1MCS-2-*pep22*, with pBBR1MCS-2-*pep23*, with pBBR1MCS-2-*pep35*, with pBBR1MCS-2-*pep51*, with pBBR1MCS-2-*pep56*, and with pBBR1MCS-2-*pep68* respectively, from left to right. (B) Photograph of agitated bacterial cultures and (C) The settled bacterial cultures with the same order as (A). (A) was visualized by light microscope (×1000 magnification); Bars: 10 μm.


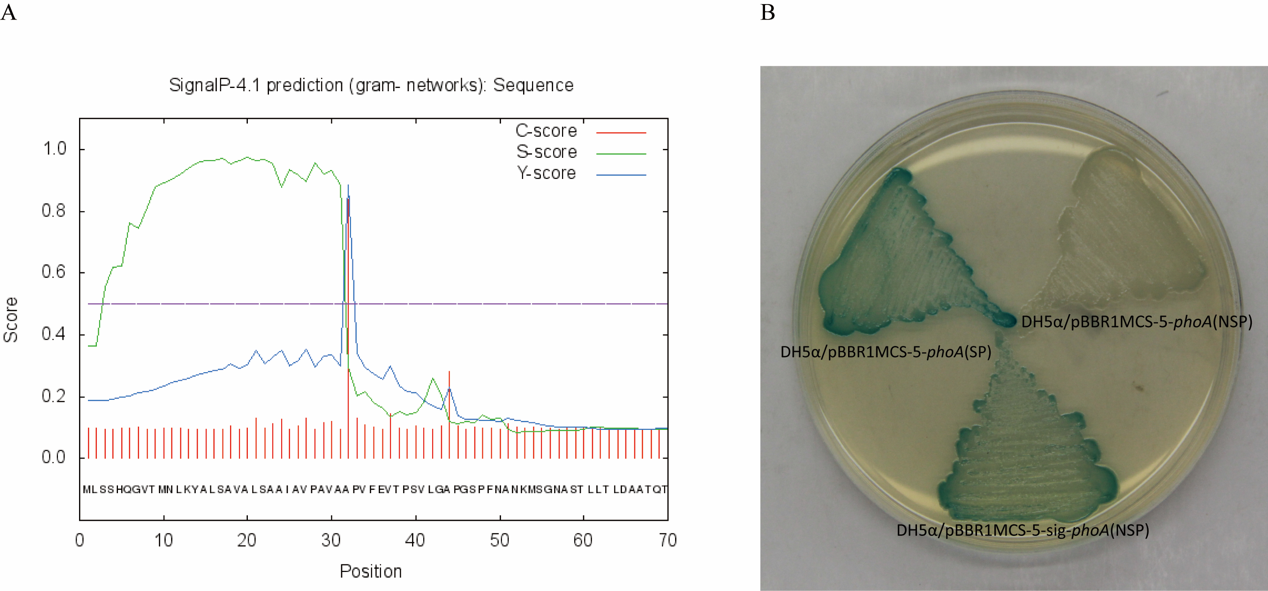


**Supplemental Figure S6** The alkaline phosphatase A (PhoA)-fusion assay demonstrated that the PEP-CTERM protein PepA is secreted into the periplasm as computationally predicted. (A) computational prediction of signal peptide for PepA secretion by using the SignalP4.0 software. The cleavage site of signal peptide is predicted to be located between amino acid residues 31A and 32A. (B) The nucleotide sequence encoding the PepA signal peptide was fused with the *E. coli phoA* gene with deletion of 5’-sequence coding for original signal peptide by using cross-over PCR. The construct pBBR1MCS-5-*phoA* expressing full-length PhoA, the colony on the left was used as positive control and the pBBR1MCS-5-phoA(NSP) (NSP stands for no signal peptide, the colony in the middle) expressing the truncated PhoA without N-terminal signal sequence as negative control.


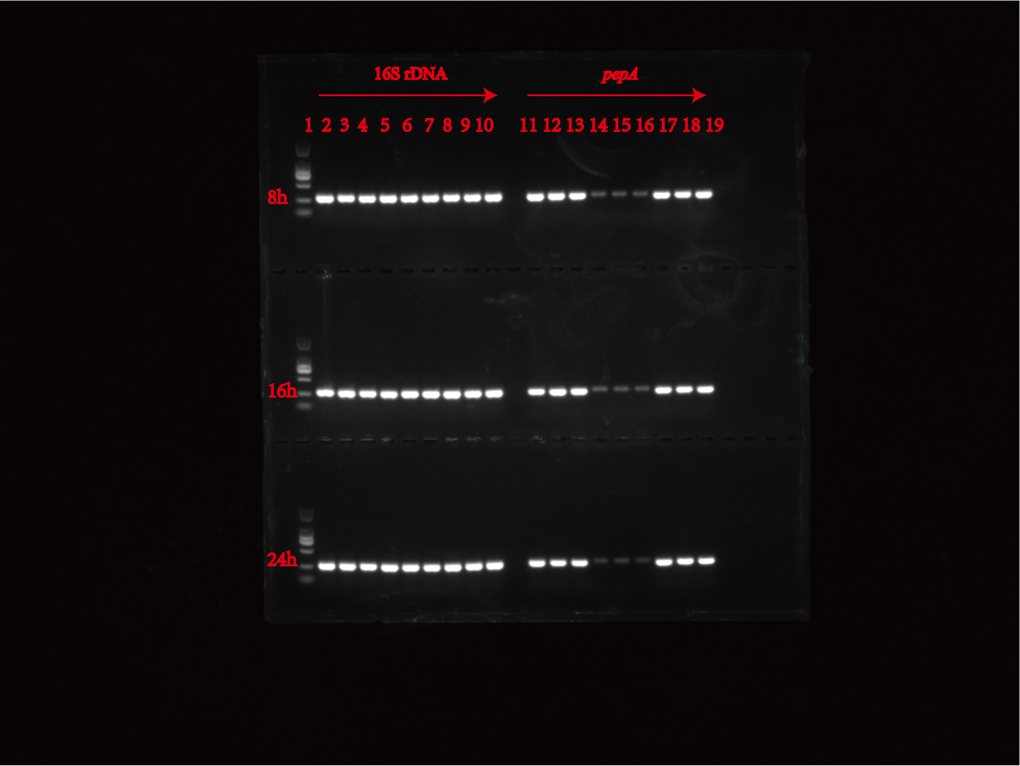


**Supplemental Figure S7** Transcription of the gene was examined by semi-quantitative RT-PCR with 16S rRNA gene as the loading control. The experiments were performed in triplicate. Lane 2-4 and lane 11-13 represented the plasmid pBBR1MCS-2 in RN12WT examined by 16S rRNA gene and *pepA* gene respectively, Lane 5-7 and lane 14-16 represented the plasmid pBBR1MCS-2 in RN12T examined by 16S rRNA gene and *pepA* gene respectively, Lane 8-10 and lane 17-19 represented the plasmid pBBR1MCS-2-*pepA* in RN12T4 examined by 16S rRNA gene and *pepA* gene respectively.

A


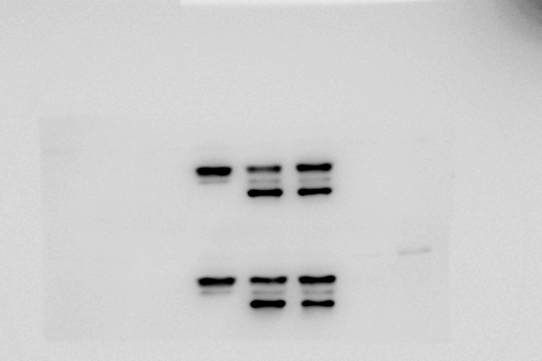

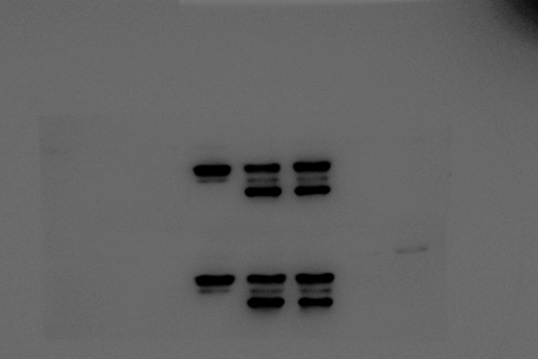


1 2 3 4 5 6 1 2 3 4 5 6

B


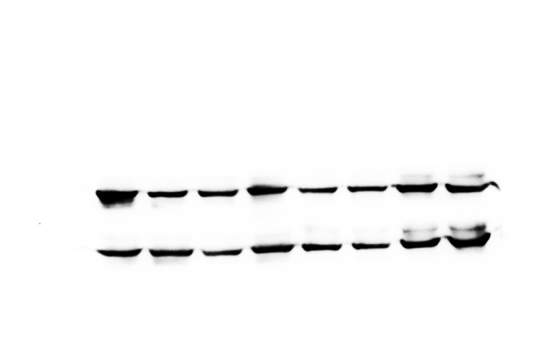

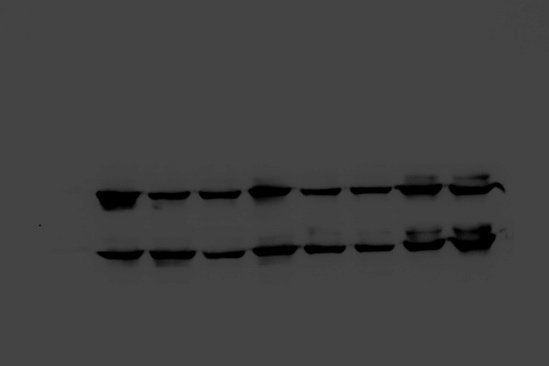


1 2 3 4 5 6 7 8 9 1 2 3 4 5 6 7 8 9

C


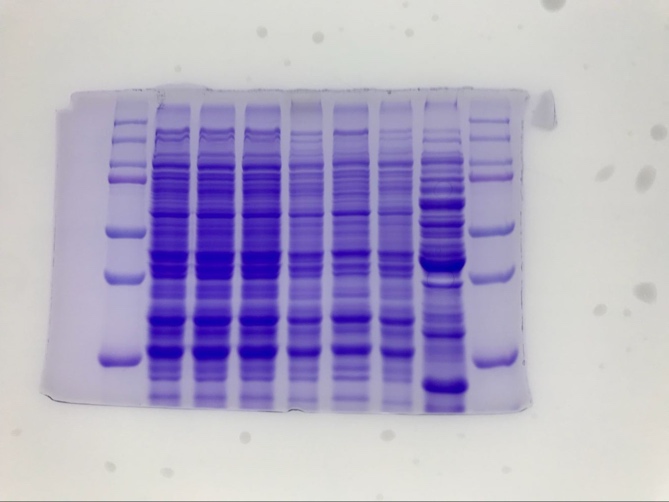


1 2 3 4 5 6 7 8 9

**Supplemental Figure S8** The full gels and blots. (A) The PVDF membrane incubated with His-tag specific antibody, lane 1 to lane 6 were the counterpart of lane 1 to lane 6 in Fig. 7. (B) The PVDF membrane incubated with anti-RpoA antibody, lane 2 to lane 7 of the lower blot were the counterpart of lane 1 to lane 6 in Fig. 7. (C) The Coomassie Brilliant Blue stain of the SDS-PAGE gel, lane 2 to lane 7 were the counterpart of lane 1 to lane 6 in Fig. 7. (A) and (B) had two replicates with different exposure conditions respectively.

1. **Livak KJ, Schmittgen TD.** 2001. Analysis of relative gene expression data using real-time quantitative PCR and the 2(T)(-Delta Delta C) method. Methods **25:**402-408.
